# Analysis of Cerebral Aneurysm Wall Tension and Enhancement Using Finite Element Analysis and High-Resolution Vessel Wall Imaging

**DOI:** 10.3389/fneur.2021.764063

**Published:** 2021-12-10

**Authors:** Adam E. Galloy, Ashrita Raghuram, Marco A. Nino, Alberto Varon Miller, Ryan Sabotin, Carlos Osorno-Cruz, Edgar A. Samaniego, Suresh M. L. Raghavan, David Hasan

**Affiliations:** ^1^Roy J. Carver Department of Biomedical Engineering, University of Iowa, Iowa City, IA, United States; ^2^Department of Neurology, The University of Iowa Hospitals and Clinics, University of Iowa, Iowa City, IA, United States; ^3^Department of Neurosurgery, The University of Iowa Hospitals and Clinics, University of Iowa, Iowa City, IA, United States; ^4^Department of Radiology, The University of Iowa Hospitals and Clinics, University of Iowa, Iowa City, IA, United States

**Keywords:** intracranial aneurysm, wall enhancement, magnetic resonance imaging (MRI), finite element analysis (FEA), wall tension

## Abstract

Biomechanical computational simulation of intracranial aneurysms has become a promising method for predicting features of instability leading to aneurysm growth and rupture. Hemodynamic analysis of aneurysm behavior has helped investigate the complex relationship between features of aneurysm shape, morphology, flow patterns, and the proliferation or degradation of the aneurysm wall. Finite element analysis paired with high-resolution vessel wall imaging can provide more insight into how exactly aneurysm morphology relates to wall behavior, and whether wall enhancement can describe this phenomenon. In a retrospective analysis of 23 unruptured aneurysms, finite element analysis was conducted using an isotropic, homogenous third order polynomial material model. Aneurysm wall enhancement was quantified on 2D multiplanar views, with 14 aneurysms classified as enhancing (CR_stalk_≥0.6) and nine classified as non-enhancing. Enhancing aneurysms had a significantly higher 95th percentile wall tension (μ = 0.77 N/cm) compared to non-enhancing aneurysms (μ = 0.42 N/cm, *p* < 0.001). Wall enhancement remained a significant predictor of wall tension while accounting for the effects of aneurysm size (*p* = 0.046). In a qualitative comparison, low wall tension areas concentrated around aneurysm blebs. Aneurysms with irregular morphologies may show increased areas of low wall tension. The biological implications of finite element analysis in intracranial aneurysms are still unclear but may provide further insights into the complex process of bleb formation and aneurysm rupture.

## Introduction

Multiple biological and mechanical phenomena regulate the process of intracranial aneurysm growth and rupture. Aneurysm growth is thought to be mediated by inflammatory factors as a response to unusual arterial mechanical stresses (1, 2). Wall proliferation associated with stable, higher flows compared to wall degradation associated with slower, diffuse flows highlight the variable response of the aneurysm wall to different patterns of wall shear stress (3). The inflammatory response to the infiltration of macrophages into the aneurysm internal elastic lamina may initiate a cycle of smooth muscle cell proliferation paired with collagen remodeling that collectively increases the aneurysm wall tension (wall stress resultant—i.e., wall stress integrated across the thickness). Maladaptation to this wall tension in addition to lipid accumulation may create focal areas in the aneurysm wall that are more susceptible to rupture (4–6).

In conjunction with mechanical parameters such as wall tension, morphological features of aneurysms including size (7), size ratio (8, 9), aspect ratio (10), and the presence of blebs (11) can provide valuable insights into an aneurysm's risk of rupture. Specifically, aneurysm morphology can create hemodynamic patterns that lead to wall remodeling or the formation of blebs (12, 13).

Recently, high-resolution vessel wall imaging (HR-VWI) has received attention as an effective, non-invasive way to study aneurysm wall dynamics and inflammatory responses (14–16). Wall enhancement may indicate increased risk of rupture in aneurysms (17–19). However, many open questions remain about the etiology of wall enhancement and its relation to aneurysm rupture and instability. It is important to investigate the interplay between wall enhancement, aneurysm morphology, and aneurysm wall tension as it relates to aneurysm growth and rupture.

Finite element analysis (FEA) can be a useful tool in simulating the mechanics of aneurysms to estimate mechanical parameters that cannot easily be measured within patients. FEA has been used to investigate the mechanics of abdominal aortic aneurysm growth and rupture (20, 21). Similar computational analyses of intracranial aneurysms have been generally limited to demonstration of the mechanics of aneurysm recurrence after endovascular treatments (22–24). By analyzing unruptured aneurysms before treatment using FEA, we aim to investigate the relationship between aneurysm wall tension, morphology and enhancement.

## Materials and Methods

### HR-VWI Image Acquisition and Subject Demographics

With IRB approval and informed consent, patients were imaged using a HR-VWI protocol on 3T MRI at the University of Iowa Hospitals and Clinics. Table 1 details the HR-VWI protocol used for the study. Twenty-seven patients with 28 total aneurysms were included.

**Table 1:** 3T HR-VWI Imaging protocol.

**Parameter**	**3D T1 SPACE**	**CE-MRA**
TR (msec)	900	3.3
TE (msec)	15	1.28
Flip angle	variable	25
Bandwidth (Hz/pixel)	446	590
FOV (mm)	200 x 200	223 x 195
Matrix (mm)	320 x 320	252 x 284
Voxel size (mm)	0.6 x 0.6 x 0.6	0.6 x 0.6 x 0.8
Slice thickness (mm)	0.63	0.8
Echo Train Length	52	0
Acquisition time	6:44	0:15

### Wall Enhancement Analysis

Wall enhancement was quantified (25, 26) from T1 post contrast images on 2D multiplanar views using the Picture Archiving Communication System, version 12.1.6.1005 (Carestream Vue PACS, Rochester, NY). Aneurysms wall enhancement was quantified using 2D regions of interest (ROIs) of the vessel wall after normalization with the pituitary stalk:


$$\begin{array}{l} CR_{stalk}=SI_{wall\;}/SI_{Pituitary\;Stalk} \end{array}$$

where the maximal signal intensity of the aneurysm wall was normalized to the maximal signal intensity of the pituitary stalk on T1 post contrast imaging. Aneurysms with a CR_stalk_≥0.6 were classified as enhancing (26).

### Image Segmentation

Three-dimensional reconstructions of 28 aneurysms were created from the best available 3DRA, CTA, or CE-MRA imaging using the Vascular Modeling Toolkit (VMTK) (27, 28). According to procedures detailed by Ramachandran et al. (29) parent vessels were reconstructed using the colliding fronts algorithm, while the aneurysm sac was reconstructed using the fast-marching algorithm with user-defined thresholds.

### Finite Element Analysis

FEA was performed to estimate the wall tension throughout the aneurysm surface. The aneurysm surface reconstructions were smoothed before FEA using the shrink wrap feature in Ansys SpaceClaim (ANSYS, Inc., Canonsburg, PA, USA) with a gap size of 0.55 mm and an angle threshold of 40°. Five aneurysms were excluded from the analysis due to poor reconstruction quality. Twenty-three aneurysms were analyzed using FEA.

We modeled the aneurysm wall as a hyperelastic (3rd order polynomial) homogenous material as described previously (30).

A static luminal pressure of 100 mmHg was applied to simulate blood pressure. Displacements were fixed on the ends of the vasculature. Ansys Mechanical (ANSYS, Inc., Canonsburg, PA, USA) was used to create and solve the FEA models. After the analyses were completed, the 95th percentile wall tension for each aneurysm was calculated from the spatial distribution of wall tension in that aneurysm model. Ninety-fifth percentile wall tension was chosen as a representative statistic over the peak wall tension to filter out stress concentrations that may have arisen from mesh artifacts.

### Statistical Analysis

Mann-Whitney U tests were used to determine if the differences in 95th percentile wall tension between enhancing and non-enhancing aneurysms were significant. Non-normally distributed continuous data were compared using Spearman's correlations while categorical data was compared using Fisher's Exact test. Additionally, a partial F-test was used to determine if enhancement was a significant predictor of wall tension while accounting for a linear effect from the aneurysm diameter. All statistics were calculated using RStudio (31).

## Results

Twenty-three patients with twenty-three aneurysms were included in the study. Fourteen aneurysms (61%) were classified as enhancing (CR_stalk_≥0.6), and nine as non-enhancing (39%). Three aneurysms (13%) were fusiform, seven aneurysms (30%) had blebs and six (26%) aneurysms were in the posterior circulation. Ninety-fifth percentile wall tension was not significantly different amongst demographic groups (Table 2). Smokers had significantly lower wall enhancement (CR_stalk_ = 0.54 ± 0.17) than non-smokers (0.86 ± 0.39, *p* = 0.032).

**Table 2:** Subject demographic information.

**Demographic**	**Total (*N* = 23)**	**95th Percentile Wall Tension (N/cm)**	***p***	**Blebs (*N* = 7)**	***p***	**Enhancement (*N* = 14)**	***p***
Women	16 (70%)	0.57 ± 0.25	0.135	4 (57%)	0.626	6 (43%)	0.176
Hypertension	11 (48%)	0.62 ± 0.28	0.652	2 (29%)	0.635	6 (43%)	1.000
Hyperlipidemia	7 (32%)	0.67 ± 0.31	0.945	2 (29%)	1.000	5 (36%)	0.648
Diabetes Mellitus	3 (13%)	0.70 ± 0.51	0.857	1 (14%)	1.000	2 (14%)	1.000
Smokers	7 (30%)	0.46 ± 0.14	0.066	3(43%)	0.334	2 (14%)	0.074

FEA was used to estimate the spatial maps of wall tension on each aneurysm under a blood pressure of 100 mmHg. The 95th percentile wall tension was then calculated for each aneurysm model. The mean (± standard deviation) 95th percentile wall tension across all aneurysms was 0.63 ± 0.28 N/cm (*N* = 23).

**Figure 1:**
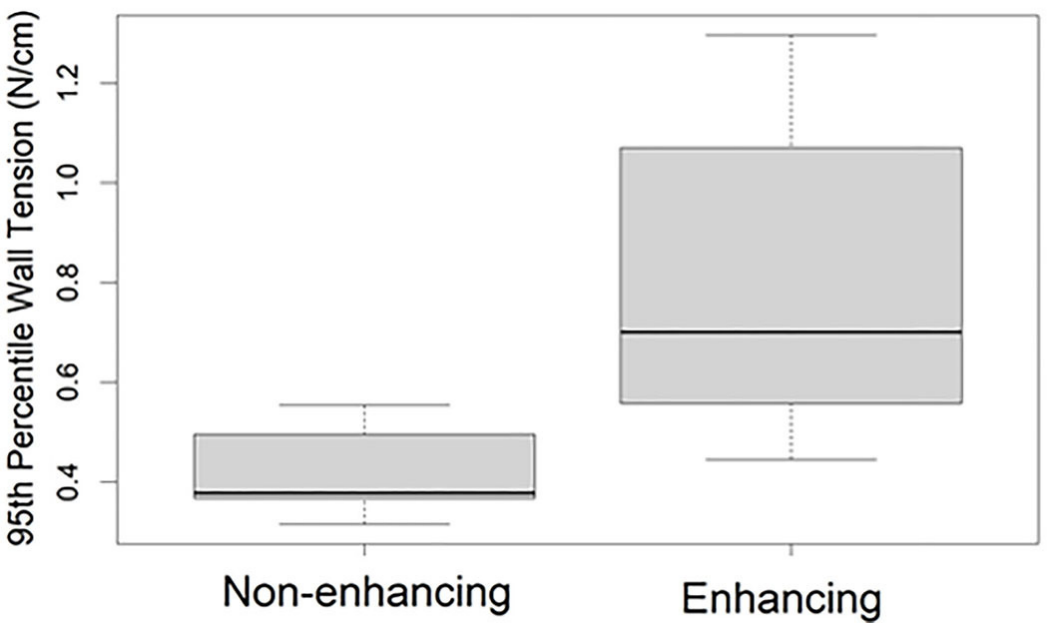
95th percentile (within subject) wall tensions in aneurysms with and without wall enhancement. The bounds of each box represent the 25 and 75th percentile wall tensions within the group, the bold line indicates the median wall tension, and the whiskers extend to the full range of wall tensions within the group.

**Figure 2:**
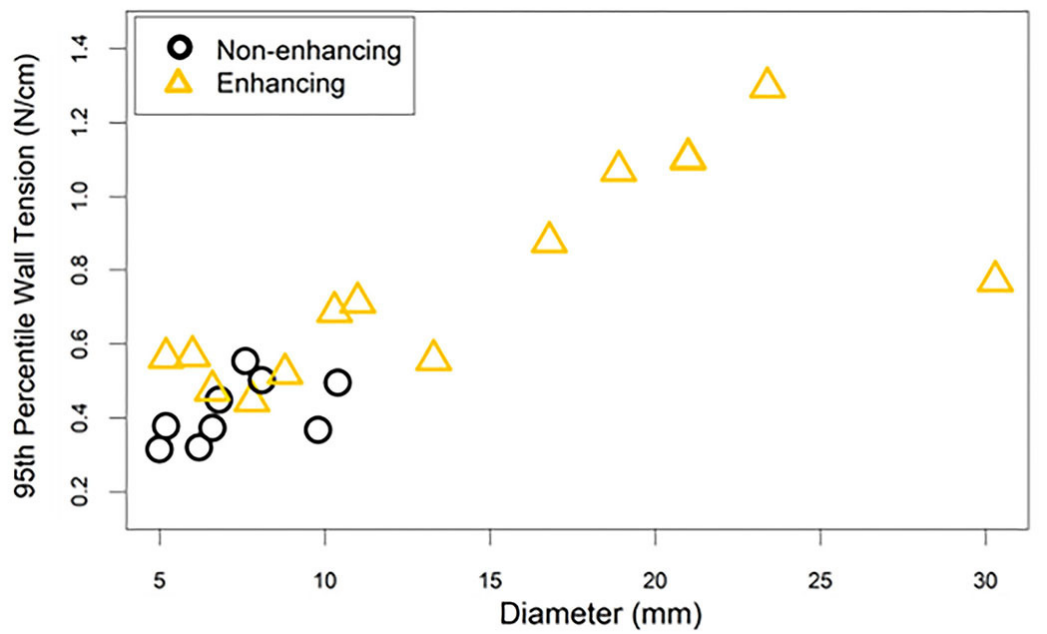
The 95th percentile wall tension for each aneurysm is plotted against the aneurysm's diameter. The correlation between diameter and wall tension must be considered when interpreting the differences in wall tension between enhancing and non-enhancing aneurysms. This is especially true considering that enhancing aneurysms tended to have larger diameters than non-enhancing aneurysms.

After stratification of the cohort into enhancing and non-enhancing aneurysms (Figure 1), the mean 95th percentile wall tensions for enhancing aneurysms (μ = 0.89 ± 0.32 N/cm) were significantly higher than non-enhancing aneurysms (μ = 0.48 ± 0.10 N/cm, *p* < 0.001). Enhancing aneurysms were significantly larger in diameter (μ = 14.3 ± 7.6 mm) than non-enhancing aneurysms (μ = 7.3 ± 1.9 mm, *p* = 0.019). Figure 2 highlights the linear relationship between 95th percentile wall tension and diameter for all aneurysms in the cohort (Spearman's *r* = 0.76). After accounting for this relationship, the F-test indicated that enhancement was a borderline significant predictor of greater 95th percentile wall tension (*p* = 0.046). 95th percentile wall tension was less strongly correlated to neck size (Spearman's *r* = 0.541), size ratio (Spearman's *r* = 0.487) and aspect ratio (Spearman's *r* = 0.206).

**Figure 3:**
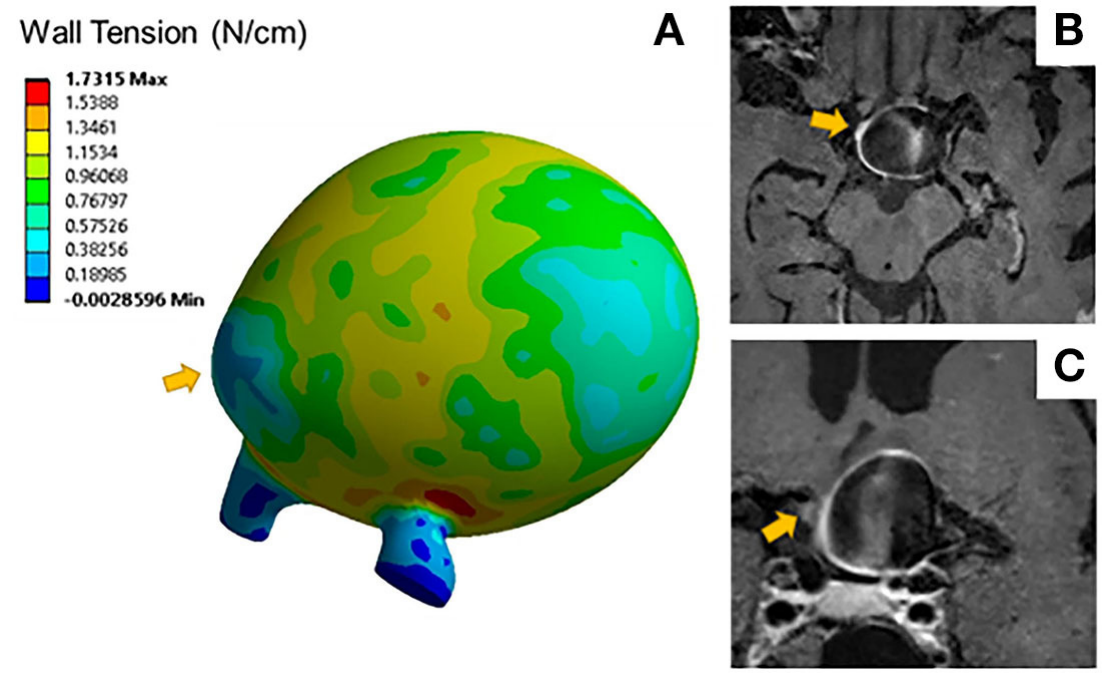
**(A)** Wall tension contours for a distal MCA aneurysm exhibits areas of low wall tension at the bleb indicated by the arrow. Sagittal **(B)** and coronal **(C)** T1 post contrast image slices of the same aneurysm show areas of focal wall enhancement at that bleb.

**Figure 4:**
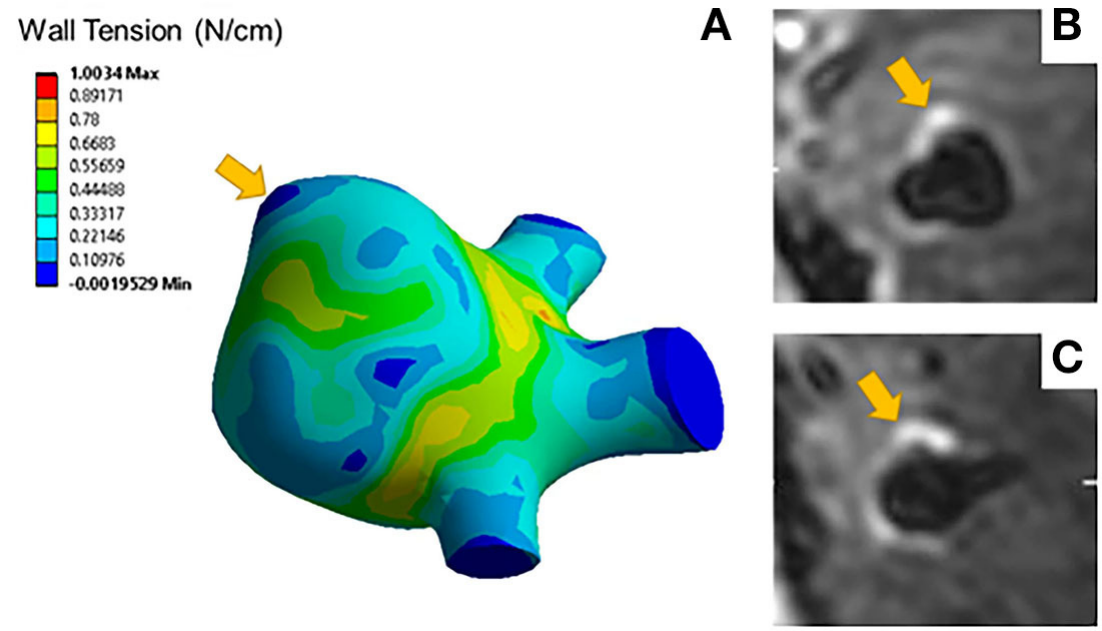
**(A)** A representative wall tension contour plot indicates elevated wall tensions at areas of low curvature such as the aneurysm neck but lower wall tension at areas of high curvature such as the area indicated by the arrow. Axial **(B)** and coronal **(C)** slices of the T1 post contrast image of the same aneurysm show focal enhancement colocalized with areas of low wall tension.

Aneurysms with blebs did not have significantly different 95th percentile wall tensions (μ = 0.51 ± 0.04 N/cm) compared to those without blebs (μ = 0.68 ± 0.32 N/cm, *p* = 0.452). Five out of seven (71%) of the aneurysms with blebs were classified as enhancing. The area of low wall tension visualized in blebs was located at the dome of the bleb, while the neck of the blebs had higher wall tension (Figure 3). Areas of low wall tension did not consistently colocalize with blebs, however some aneurysms with focal enhancement showed locally low wall tension in areas of high curvature (Figure 4).

## Discussion

Results from FEA showed significantly greater peak wall tensions in enhancing aneurysms compared to non-enhancing aneurysms. Aneurysm size, a known predictor of rupture (7), has been found to be independently associated with wall enhancement (32–34). In addition, according to the Law of Laplace, we would expect increasing wall tension with increasing aneurysm radius (35). When accounting for a linear effect from diameter on wall tension, enhancement remained a significant predictor of wall tension. This result suggests that the relationship between wall enhancement and wall tension that we observed arises primarily from the differences in size between the two groups, but additional relationships may exist. However, because of our study's small sample size and the skewed distribution in diameters between enhancing and non-enhancing groups (Figure 2), some caution must be taken in interpreting these results.

Wall tension is highly dependent on geometric features; it can describe patterns of aneurysm rupture and growth due to irregular morphology that may lead to the mechanical loading present before the inflammatory response described by Frosen et al. (4) In this study, we did not find other morphological indicators of instability such as size ratio, aspect ratio, or the presence of blebs to be correlated with increased wall tension, but we may be limited by the small amount of aneurysms (*N* = 7) with irregular morphologies or blebs.

In an analysis of the effect of morphology and material model on wall tension in intracranial aneurysms, Lu et al. (36) found that peak wall tension is highly dependent on aneurysm geometry, and likely to concentrate in saddle regions with low curvature, such as the aneurysm neck or bleb transition zone. Our results support the expected patterns of wall tension (Figure 3A), with low wall tension concentrating at areas of high curvature, such as aneurysm blebs. The mechanical impetus for wall remodeling that leads to the formation of blebs may be related to both the aneurysm geometry and hemodynamic flow patterns (37). The effects of size and tissue elasticity on flow velocity profiles may create the unique patterns of wall shear stress attributable to aneurysm growth and rupture (4, 38). Robertson et al. investigated the loading effects on collagen organization in 15 unruptured aneurysms after surgical clipping, suggesting that rupture mechanics may be related to differences in wall structure and remodeling (39). In this study, increased wall tension was consistently concentrated at the neck, and reduced wall tension was concentrated at the dome. Further studies are required to accurately characterize the relationship between these forces and the previously described hemodynamic forces that may provoke subsequent growth and rupture.

Focal wall enhancement (Figures 4B,C) may be similarly connected to this complex remodeling process (40, 41). Understanding enhancement as a biomarker for inflammation, focal enhancement may reveal areas more prone to rupture due to macrophage infiltration related to the regional differences in wall tension. Without histological samples, however, we cannot definitively identify this relationship in our study.

In addition to small sample sizes, some limitations in the FEA should be considered. The FEA models assumed uniform wall thickness and material properties between and within each aneurysm. Precisely for this reason, we have focused on wall tension (stress resultant, not stress) because it is insensitive to wall thickness, wall material model, and parameters (30). However, this focus on wall tension obviated our ability to study important associations between regional variations in wall stress and wall structure (13, 42). Using advanced imaging techniques, Niemann et al. (43) have made great advances in using histological samples to create patient specific 3D models of aneurysm walls with heterogeneous structure. Incorporating heterogenous wall properties into a patient specific FEA model noninvasively remains a challenge.

In summary, enhancing aneurysms had significantly greater peak wall tension than non-enhancing aneurysms. This relationship partially arises from the fact that enhancing aneurysms have larger sizes than non-enhancing aneurysms. An additional correlation between wall tension and diameter was identified when controlling for aneurysm size, but our small sample size suggests interpreting these results cautiously. Although small sample size limits our study's conclusions, our approach of combining computational models with HR-VWI is promising for isolating specific mechanical factors that may influence aneurysm wall remodeling and relating them to imaging features visible to a clinician.

## Data Availability Statement

The raw data supporting the conclusions of this article will be made available by the authors, without undue reservation.

## Ethics Statement

The studies involving human participants were reviewed and approved by University of Iowa IRB. Written informed consent for participation was not required for this study in accordance with the national legislation and the institutional requirements.

## Author Contributions

ES, SR, and DH conceived of the project and provided guidance on its completion. AR, AV, RS, and CO-C participated in data selection. AR, MN, and AV analyzed and processed the medical images. AG performed computational mechanics analysis. AG, AR, MN, AV, RS, CO-C, ES, SR, and DH participated in the statistical analysis and interpretation of data. AG and AR wrote the first draft of the manuscript. AG, AR, MN, AV, RS, CO-C, ES, SR, and DH read the manuscript, provided feedback for revisions, and approved the final version. All authors contributed to the article and approved the submitted version.

## Conflict of Interest

The authors declare that the research was conducted in the absence of any commercial or financial relationships that could be construed as a potential conflict of interest.

## Publisher's Note

All claims expressed in this article are solely those of the authors and do not necessarily represent those of their affiliated organizations, or those of the publisher, the editors and the reviewers. Any product that may be evaluated in this article, or claim that may be made by its manufacturer, is not guaranteed or endorsed by the publisher.
